# Tissue integrity impacts of application of 160–200 kJ of 915 MHz microwave energy, using the DTS: Diathermic Syncope® system, to the forehead of cattle, and alignment with the requirements of religious slaughter markets

**DOI:** 10.1016/j.vas.2025.100464

**Published:** 2025-06-04

**Authors:** Alison Small, Ian Jenson, Bruno Fiszon, Pierre Le Neindre, Andrew Phillips, David McLean, Joel McLean, Troy Kalinowski, James Ralph

**Affiliations:** a FD McMaster Laboratory, Agriculture and Food, CSIRO, Locked Bag 1, Armidale NSW 2350, Australia; b FIRST Management Pty Ltd, Barramattigal Country, PO Box 2523, North Parramatta NSW 1750, Australia; c Chief Rabbi of Metz, Doctor of Veterinary Medicine, 39 Rue Elie Bloch, 57000 Metz, France; d INRAE, Rue de l′université DEPE, 147 Rue de l′Université, 75007 Paris, France; e Wagstaff Food Services Pty Ltd, 1500 Thompson Road, Cranbourne VIC 3977 Australia; f Advanced Microwave Technologies, 34 Dobbie Avenue, East Corimal NSW 2518, Australia

**Keywords:** Humane slaughter, Beef, Welfare, Histology, Diathermic syncope, Stunning, Electromagnetic

## Abstract

Although society desires good animal welfare at slaughter, there is a tension between some religious requirements and modern commercial stunning methods. The DTS (Diathermic Syncope®) system has been designed with a vision of relieving that tension, rendering the animal unconscious within 0–5 s, by applying 160–200 kJ of 915 MHz electromagnetic energy to the forehead, while limiting damage or changes to the tissues. To investigate this latter aspect, surface temperature at the point of application was measured using fiber optic sensors in 12 animals; application point hide skin of 3 animals was examined histologically; 7 brains were inspected and palpated shortly after energy application; and four brains were examined histologically. There were no gross morphological changes to the brain, and limited evidence of changes on histological examination. Temperatures at the point of application remained close to the ambient (29.0–36.5 °C) prior to and for the first 8–12 s of energy application (max duration of application 12 s), then rose steadily to a peak, and began to fall when energy application ceased. Maximum recorded temperature was 54.27 (± 6.56) °C, which occurred after energy application had ceased. Although small numbers of animals were included in this study, they and the process used were representative of the normal operation through the commercial abattoir. Depilation and effects on forehead skin noted on carcases after bleed-out are likely to be exacerbated by post-mortem change. These findings may comply with religious slaughter requirements.

## Highlights

• Application of 160–180 kJ energy to the forehead resulted in no visible damage to brain tissue.
• Minor histological changes in brain could be associated with processing artefact.
• Forehead temperature remained close to ambient for the first 8 – 12 s of energy application (max duration of application 12 s), then rose steadily to a peak, and began to fall when energy application ceased.
• Depilation and skin changes noted on carcases after bleed-out are likely due to retained heat and are likely to occur post-mortem.
• Minimal impacts on brain and skin tissue may comply with religious slaughter requirements.

## 1 Introduction

A dielectric (electromagnetic) system for the induction of unconsciousness in cattle has been developed by Wagstaff Food Service Pty Ltd and Advanced Microwave Technologies. In brief, this system, trademarked DTS: Diathermic Syncope® (DTS), delivers 160–180 kJ of electromagnetic energy, at a frequency of 915 MHz and an incident power of 16–18 kW to the forehead of cattle that are restrained in box fitted with a chin lift. Loss of consciousness is induced within 0–5 s of onset of energy application, and insensibility is of sufficient duration, confirmed by electroencephalogram (EEG), to allow exsanguination prior to recovery (McLean et al., 2017; Musk & Johnson, 2024; Small et al., 2019). The physiological responses and post-slaughter meat quality in treated cattle were the same as those stunned using penetrative captive bolt (Small & Hughes, 2022).

DTS has the potential to meet Halal and Kosher requirements, without current disadvantages of existing stunning methods (e.g., - the potential for cracked skulls associated with percussive stunning; - the need to use an electro-immobilizer after stunning to suppress convulsive movements allow the neck cut to be performed safely in electrical head-only stunning; - the need for a second exsanguination cut following the neck cut to remove the risk of the animal regaining consciousness during bleed-out). In fact, even the term ‘stunning’ has negative connotations in the Kosher and Halal traditions, as the verb ‘to stun’ means ‘to make senseless, groggy or dizzy by or as if by a blow’ (Merriam-Webster, 2025), which suggests an episode of violence towards the animal. Violence in any form is not condoned, and the expectation is that all species are treated with kindness (Anil et al., 2010; Foltz, 2006; Kumar et al., 2023; Masri, 2007; Solomon, 2009; Zivotofsky, 2010; Zürek et al., 2021).

For DTS to be acceptable for Halal or Kosher meat many requirements must be met, only some of which are relevant to pre-slaughter insensibility. For both Halal and Kosher slaughter there is an expectation that the animal is treated with respect, and that the whole process does not cause distress or anguish to the animal (Riaz et al., 2021; Zivotofsky, 2010). Furthermore, the animal must be alive and healthy immediately before the cut is made (Riaz et al., 2021; Sazili et al., 2023; Zivotofsky & Strous, 2012). Currently, no method of stunning is acceptable for Kosher slaughter due to the fact that all result in some form of harm or damage to the animal, and it is a requirement that the animal remains alive and healthy in the period between stunning and cutting (Zivotofsky & Strous, 2012). Most Halal slaughter occurs after stunning, with the expectation that the stunning is reversible and the animal able to regain full consciousness (Levinger, 1995; Riaz et al., 2021; Sazili et al., 2023). Both Halal and Kosher slaughter require the animal to die from exsanguination (Sazili et al., 2023; Zivotofsky & Strous, 2012). There is an expectation in Kosher slaughter that there are jerky movements of the limbs post-slaughter as a sign that the animal was alive when slaughtered (Zivotofsky, 2023). For Kosher meat there is a concern for numerous physical defects in the carcase and organs (Levin & Boyden, 1940), and as a consequence some animals, that would be considered fit for consumption under secular food safety legislation, are not entering the Kosher market. A concern with electrical stunning is that it may induce defects that are difficult to detect and may mask others (Zivotofsky, 2010) and that the rise in brain temperature induced by DTS could lead to unacceptable physical damage (Chao, 2022; Farouk et al., 2015; Fuseini et al., 2017; Nakyinsige et al., 2013; Regenstein et al., 2003; Riaz et al., 2021; Zivotofsky, 2023).

Some religious requirements, such as the animal being alive at the time of the neck cut and able to recover from the stun are well known. Observations of the brain would contribute to an assessment of the potential of DTS to induce defects in the animal. Observations of the skin at the point of DTS application would provide an understanding of the nature of the damage that may be caused and the potential impact on animal health and well-being. To contribute to bridging the gap between the commercial and animal welfare expectations of secular markets and those of religious requirements, this paper reports on measurements of forehead temperature during DTS application and examinations of brain and skin tissue at the site of DTS application in a cross-section of slaughter-aged cattle.

## 2 Materials and methods

### 2.1 Animal ethics

This study was conducted under the authority of the CSIRO ‘Wildlife and Large Animal’ Animal Ethics Committee, reference 2021–05, in accordance with the provisions of the Australian Code for the care and use of animals for scientific purposes (National Health & Medical Research Council, 2013).

### 2.2 Description of the DTS: diathermic syncope® system

**Fig. 1:**
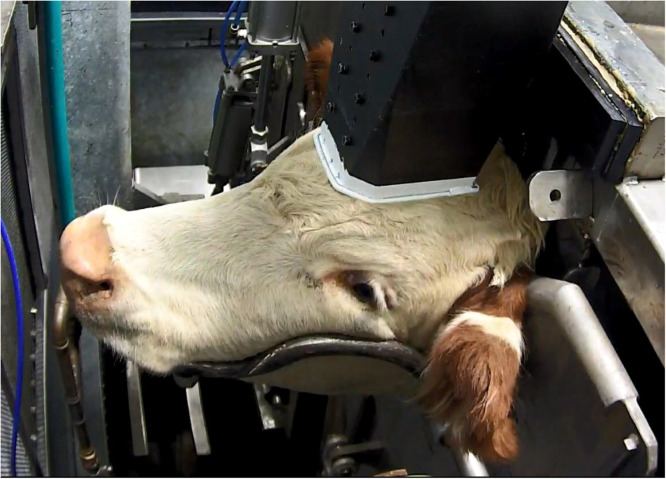
Animal’s head captured in the chin-lift, with waveguide in place, immediately prior to DTS energy application.

In the abattoir, cattle are restrained in a box fitted with neck capture and chin lift, designed to minimise movement of the head (Fig. 1). The waveguide applicator is positioned midline on the flat surface of the forehead, between the eyes and ears, forward of the poll. Positioning is facilitated by a rubber stopper or ‘datum point’ at the rear of the poll, such that when the waveguide rests against this stopper, it is in the correct position. From neck yoke capture to waveguide in position takes 30–90 s. The entire restraint box and animal is surrounded by a faraday cage to protect operators in the event of energy leakage. The doors on the faraday cage are secured with electromagnetic locks, which are linked to the control system software such that if the locks are not active, energy will not flow. Energy is generated by a magnetron housed separately and transferred to the waveguide via an autotuner that minimises reflected energy and optimises efficiency of energy transfer into the brain. The system has undergone significant refinements and optimisation since the previously published data, and consistent application of energy has been achieved. At 18 kW, 160 kJ of energy are delivered over a 10 s period, 180 kJ over 11 s and 200 kJ over 12 s, including the generator-start-up sequence, and the animal enters an unconscious state, based on the presence of a tonic-clonic response, temporary loss of rhythmic breathing and absence of eye reflexes. between 0 and 5 s after the onset of energy delivery.

### 2.3 Temperature changes at the point of application

**Fig. 2:**
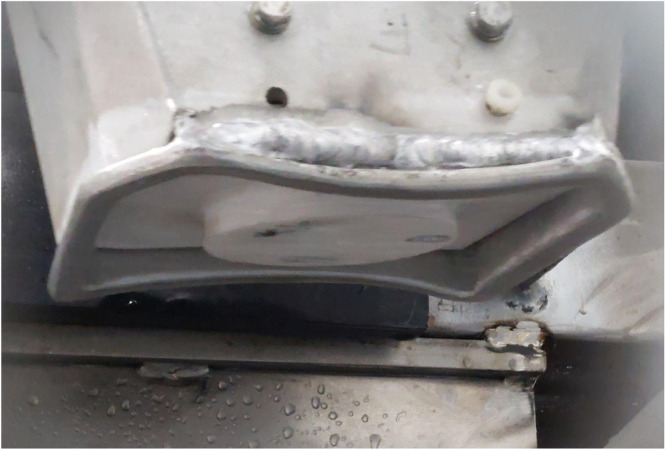
Teflon insert within applicator.

Fiber optic temperature sensors (FISO Technologies, Quebec QC, Canada) were inserted into a Teflon plate at the tip of the applicator, where it contacts the forehead of the animal (Fig. 2). The sensors were fed through a hole in the applicator wall and guided through individually drilled holes in the insert, ensuring a secure fit. To improve probe-to-skin contact, the holes were scalloped out. For each trial, the Teflon insert was firmly pressed against the bovine head to maintain consistent positioning and contact. The use of fiber optic probes enabled continuous, uninterrupted recording of temperature rise within the microwave field without being affected by electromagnetic noise. Data was captured and plotted using FISO Commander (FISO Technologies, Quebec QC, Canada) at a sampling rate of one sample every 0.45 s. Each trial utilised three probes simultaneously, providing localised temperature readings throughout the energy application process. Temperature recording ended when the waveguide was lifted from the forehead of the unconscious animal, and the animal was moved to the exsanguination point and processed for human consumption.

Twelve Australian Angus steers, estimated liveweight 430–500 kg, were used for this trial, conducted on a single day in February 2025. The first three received 200 kJ energy at 18 kW, and the temperature data was inspected prior to continuing the trial. At this stage it was decided to closely clip the hair of the forehead of the remaining animals to minimise any insulating effect of the hair coat, and to adjust the tips of the sensors to protrude 1–2 mm from the surface of the Teflon plate. Three further animals received 200 kJ, three received 180 kJ and 3 received 160 kJ, all applications at 18 kW power.

### 2.4 Effects on skin at the DTS application point

Three skin pieces from a single batch of Hereford cross animals (carcase weights not recorded) processed using 180 kJ energy application, at 18 kW power in September 2023 were evaluated. They had been excised from the cattle heads approximately 30 min after DTS energy application, during carcase dressing, refrigerated, and shipped with ice packs, arriving at the laboratory 4 days after slaughter. Animal H1 had been processed with no prior preparation; Animal H2 had the hair clipped close (no cover comb attached to the clipper blades); Animal H3 had a water-soaked hide piece laid over the top of the forehead, under the applicator aperture, such that the area remained thoroughly wetted and steam could be retained in the area during DTS application. Three punch biopsies were collected from each skin piece: one from the centre of the application area, one from the margin of the application area, and one approximately 4 cm outside the application area. Samples collected were full-thickness and ranged from 9 mm to 14 mm thick. Samples were immediately placed in histology cassettes (Techno-Plas, St Marys SA, Australia) and immersed in 10 % neutral buffered formalin solution (Hurst Scientific, Forrestdale WA, Australia). After 7 days they were rinsed twice in 80 % ethanol solution (ChemSupply Australia, Port Adelaide SA, Australia), then stored in 80 % ethanol solution until processed. Stored samples were embedded in wax, sliced and mounted, then stained with Haematoxylin and Eosin (H&E) for evaluation by light microscopy.

### 2.5 Effects on brain following DTS application

The skulls of four Murray Grey cattle, estimated liveweight 350–500 kg, in a single batch processed in August 2022, with energy delivered at 180 kJ and 18 kW, were opened to allow inspection of the brain, approximately 45–60 min post energy application (after carcase processing) and three samples, each 5 mm x 5 mm x 5 mm were collected from each brain: one from the cerebral cortex close to the poll, approximately 2 cm from the cerebellum, one from the application point, approximately 3 cm from the proximal tip of the cerebral cortex, and one from the underside of the brainstem, in the region of the medulla oblongata. All samples were no more than 5 mm from midline. Samples were immediately placed in histology cassettes and processed as described above.

A second assessment was carried out in February 2025, in which a further seven brains from a single batch of Australian Angus steers, estimated liveweight 400–530 kg, were visually inspected and palpated shortly after slaughter. Four were from cattle that had received energy at 160 kJ, one at 180 kJ, one at 200 kJ and one at 220 kJ. All applications were at 18 kW power. Skulls were opened between 10 and 45 min post energy application, dependent on availability of the bandsaw operator.

## 3 Results

### 3.1 Temperature changes at the point of application

**Fig. 3:**
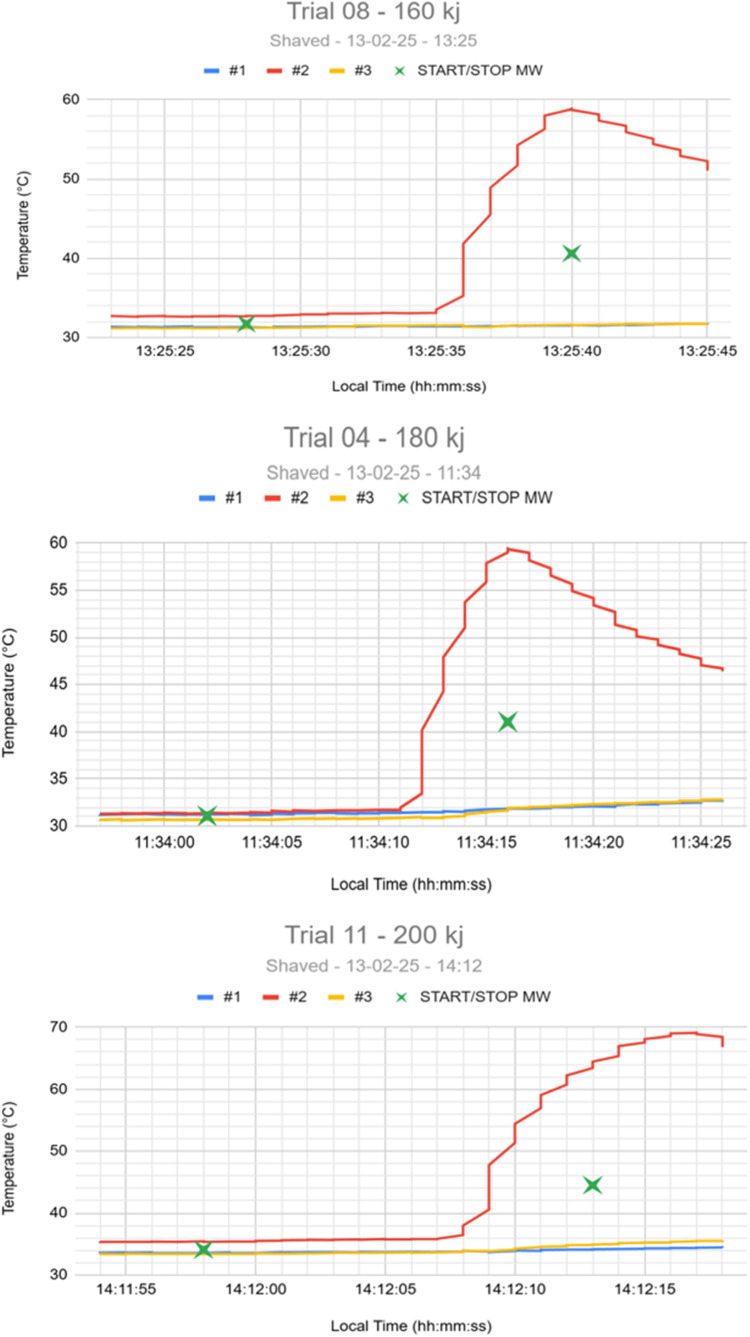
Examples of temperature profiles from individual animals (trials) receiving 160 kJ (top), 180 kJ (middle) and 200 kJ (bottom). Green stars indicate onset and offset of energy application.

Baseline temperatures prior to application were in the range of 29.0–36.5 °C, reflecting the change in ambient temperature as the day progressed (min–max 18.1–36.4 °C, Australian Bureau of Meteorology Nilma North station, recorded approximately 27 km distant). Only one of the three probes (probe 2) consistently recorded a significant temperature rise, increasing from around 30 °C up to a maximum of 54.27 (± 6.56) °C, while the others registered only minor changes (Fig. 3). Further investigation indicated that the particular applicator in use was generating a hotspot at probe 2, as opposed to the more uniform heating produced by previous applicators (data not shown). Thus, the readings at probe 2 could be considered a worst-case scenario, and therefore the data for probe s 1 and 3 were discarded for the current analysis. The temperature rise detected at probe 2 began slowly then increased in rate between 8 and 12 s after onset of energy delivery (Table 1). Maximum temperature was reached after energy delivery had been completed in all cases, and the maximum temperature recorded in any application was 69.1 °C (animal 11, 4 s after completion of energy application.

**Table 1:** Mean (± *s*.d.) temperatures °C (*n* = 3 animals in each group) by second from onset of energy application. Double line in each column indicates the end of energy application.

Time from onset of energy application	160 kJ Hair clipped	180 kJ Hair Clipped	200 kJ Hair clipped	200 kJ Hair not clipped
Baseline	31.92 (± 2.47)	32.73 (± 1.17)	34.98 (± 1.24)	29.78 (± 0.36)
1 s	31.96 (± 2.47)	32.72 (± 1.15)	35.02 (± 1.23)	29.83 (± 0.35)
2 s	32.08 (± 2.45)	32.80 (± 1.18)	35.12 (± 1.25)	29.88 (± 0.40)
3 s	32.12 (± 2.45)	32.89 (± 1.20)	35.24 (± 1.30)	29.96 (± 0.33)
4 s	32.20 (± 2.44)	33.03 (± 1.28)	35.33 (± 1.27)	30.11 (± 0.22)
5 s	32.24 (± 2.44)	33.13 (± 1.35)	35.44 (± 1.29)	30.24 (± 0.21)
6 s	32.28 (± 2.42)	33.16 (± 1.35)	35.50 (± 1.27)	30.36 (± 0.21)
7 s	32.38 (± 2.49)	33.18 (± 1.36)	35.63 (± 1.45)	30.43 (± 0.20)
8 s	33.68 (± 3.36)	33.20 (± 1.34)	35.73 (± 1.52)	30.48 (± 0.20)
9 s	38.50 (± 6.92)	33.37 (± 1.33)	36.22 (± 1.77)	30.52 (± 0.18)
10 s	43.95 (± 8.30)	35.42 (± 1.51)	37.93 (± 3.85)	30.60 (± 0.23)
11 s	47.13 (± 10.23)	42.76 (± 6.30)	41.48 (± 9.00)	30.64 (± 0.20)
12 s	48.43 (± 11.14)	47.47 (± 8.29)	44.80 (± 10.31)	30.68 (± 0.22)
13 s	48.03 (± 10.72)	52.66 (± 6.51)	47.72 (± 11.10)	30.73 (± 0.21)
14 s	47.00 (± 9.96)	54.27 (± 6.56)	49.28 (± 12.00)	30.75 (± 0.21)
15 s	46.02 (± 9.23)	53.53 (± 6.08)	49.88 (± 12.93)	30.73 (± 0.18)

### 3.2 Effects on skin at the DTS application point

**Fig. 4:**
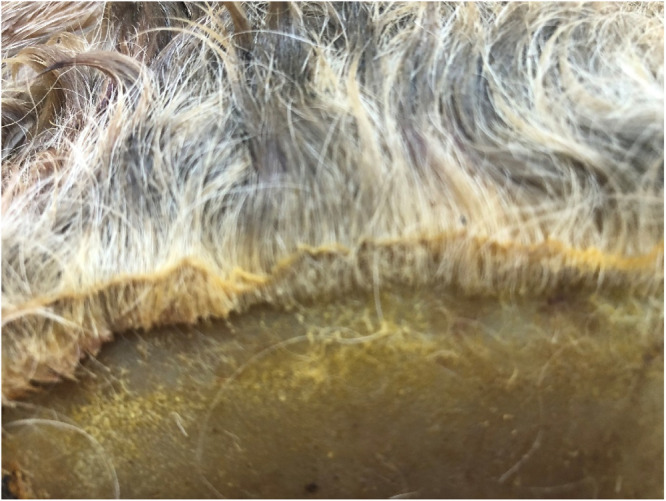
Edge of depilated area from Animal H1. A line of suint can be seen in the hair adjacent to the depilated area.

On visual inspection, the applicator footprint was evident as an area of hair loss (depilation) on all three skin pieces. The depilated area was flexible and rubbery in texture. At the edge of the depilated area, the suint (grease and dandruff) could be seen lifting away from the skin surface (Fig. 4). The underside of the pieces appeared normal, with visible connective tissue and intact blood vessels.

**Fig. 5:**
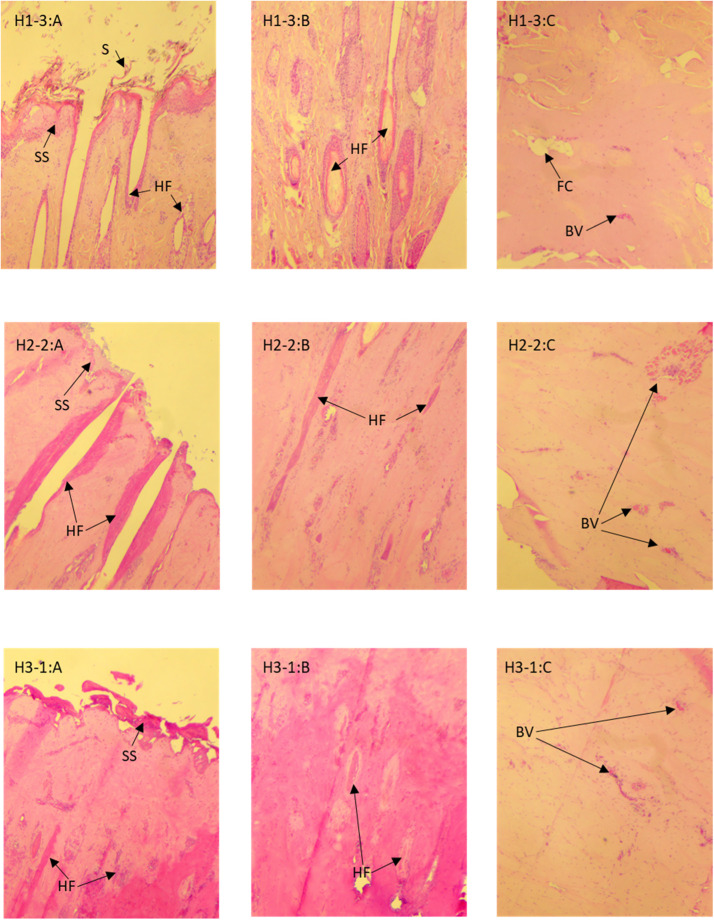
Histological views of bovine skin tissue (40x magnification) after DTS application (180 kJ, 18 kW). Horizontal: A – epidermal layer; B – papillary layer of the dermis; C – reticular layer of the dermis. Vertical: H1–3 – hide sample 1, biopsy 3 (remote from application point); H2–2 – hide sample 2, biopsy 2 (margin of depilated area); H3–1 – hide sample 3, biopsy 1 (centre of depilated area). BV – blood vessel; FC – fat cell; HF – hair follicle; S – suint; SS – stratum spinosum of epidermis.

Histology slides were assessed with reference to standard histology textbooks (Ross et al., 1989; Wheater et al., 1979). The fixation process had desiccated the samples, making them brittle and challenging to cut cleanly. Nevertheless, the structures within the tissue were clearly identifiable. From each slide, three areas were examined and photographed: the epidermis (top surface); the papillary layer of the dermis (within 2 mm from the surface) and the reticular layer of the dermis (5–8 mm from the surface). Fig. 5 shows examples of the images captured, with biopsy 3, remote from the application point at the top as a reference sample. In biopsy 3, normal epithelium (SS) is present on the surface of the biopsy (top left), with suint visible above the surface (S). Hair follicles (HF) and the cellular structure of their linings are clearly visible in the dermis (top left and top centre), while fat cells (FC) and blood vessels (BV) can be seen in the reticular layer (top right).

At the margins of the application point (middle row, hide piece 2 as the example), there is patchy loss of the epidermis (middle left), and the connective tissue supporting the cells in the dermis is less well defined (centre). However, the reticular layer (middle right) appears unchanged, and intact blood vessels and red blood cells are evident.

In the centre of the depilated area (bottom row, hide sample 3 as the example), there is more marked loss of the epidermis (bottom left), and further coagulation of the tissues of the papillary layer of the dermis (bottom centre), with loss of definition around the remaining hair follicles. However, the reticular layer (bottom right) appears unchanged, and intact blood vessels and red blood cells are evident. Subjectively, Hide sample 3 (in which moisture and steam were trapped between the applicator aperture and the forehead) appeared to have greater epidermal loss than samples 1 and 2.

### 3.3 Effects on brain following DTS application

**Fig. 6:**
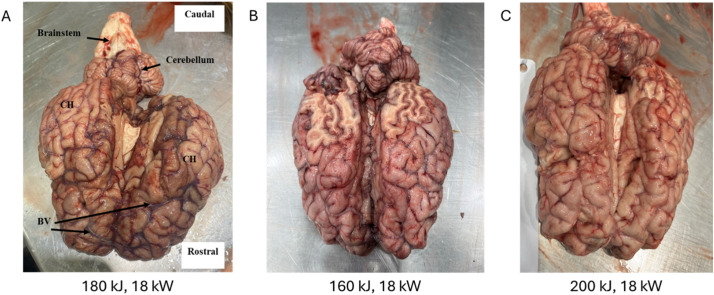
A: Bovine brain after DTS application (180 kJ, 18 kW), harvested 45 min after energy application. CH: cerebral hemisphere; BV: blood vessel. Hyperaemia and blood vessel engorgement is visible on the rostral parts of the cerebral hemispheres. B: Bovine brain after DTS application (160 kJ, 18 kW), harvested 10 min after energy application. The caudal surface of the cerebral hemispheres was accidentally removed by the bandsawing process opening the skull. C: Bovine brain after DTS application (200 kJ, 18 kW), harvested 10 min after energy application.

On gross inspection there was no physical damage to brains from 160 kJ, 180 kJ and 200 kJ applications. Brains that had been harvested with a delay of 30–45 min from energy application showed superficial hyperaemia and engorgement of blood vessels on the rostral parts of the cerebral hemispheres (Fig. 6). This was not evident on brains that were harvested within 10 min of energy application. The brain from the 220 kJ application showed disruption of the meninges covering the brain and liquefaction of the superficial tissues.

**Fig. 7:**
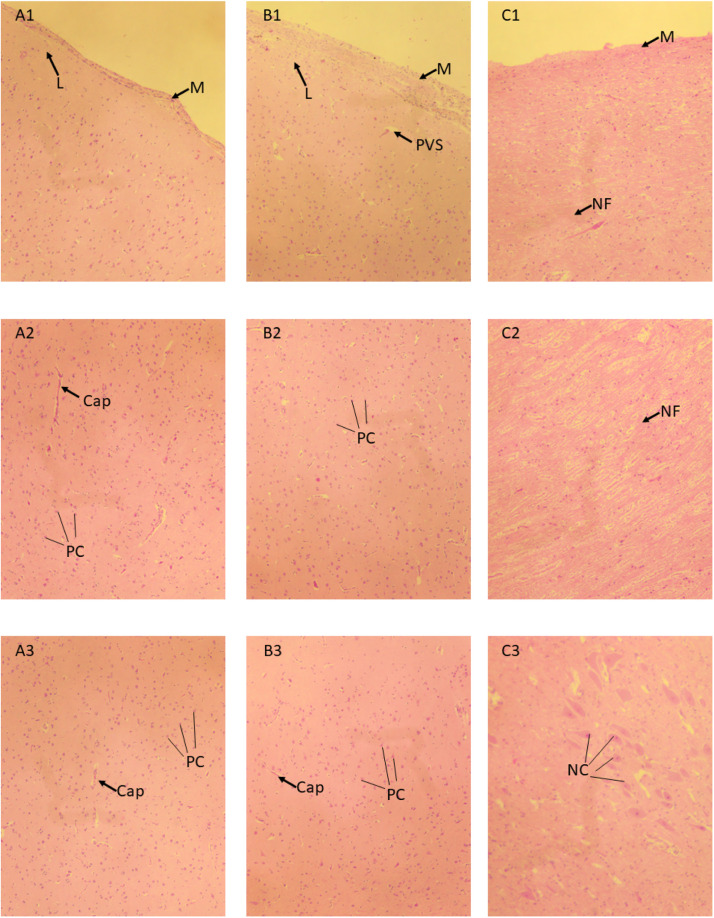
Histological views of bovine brain tissue (40x magnification) after DTS application (180 kJ, 18 kW). Vertical: A – dorsal surface of cerebrum, approximately 2 cm rostral of cerebellum; B – dorsal surface of cerebrum at application point, approximately 2 cm from rostral tip of brain; C – ventral surface of brainstem below cerebellum. Horizontal: 1: surface; 2: 2 mm depth; 3: 4 mm depth. Cap – capillary; L – lucency; M- meninges; NC – nerve cell; NF – nerve fibre; PC – pyramidal cell; PVS – perivascular space.

Histological evaluation of brain samples from the four animals processed at 180 kJ showed minimal changes to tissue (Fig. 7). Meninges (arachnoid mater and pia mater) were intact on the surface of each sample and within sulci, there was no evidence of malacia within the tissues or coagulation of brain tissue or blood within subarachnoid or penetrating vessels. Small areas of lucency were evident immediately below the pia mater in some fields of view of the cerebral surface (e.g., B1 in Fig. 7). It is unclear whether these are a result of processing artefact, or a result of the energy application. Brainstem tissue appeared normal.

## 4 Discussion

Based on a review of previously published literature on the development of the DTS system, Zivotofsky (2023) expressed concerns about lasting damage to brain tissue, due to the thermal tolerance of brain cells. The current study failed to demonstrate significant damage to brain tissue on both a gross and histological level, provided that energy applications were within the 160 – 200 kJ range. This is likely to be a result of the short duration of heating resulting from using the DTS system, there being a time-temperature relationship in the effects of heat on both eukaryotic and prokaryotic cells (Goulter et al., 2008; Knapp & Huang, 2022). It appears that the maximum thermal tolerance of nervous tissue lies in the range of 40–60 min at 42 °C, or 10–30 min at 43 °C (Fike, Gobbel, Satoh, & Stauffer, 1991, Lyons, Britt, & Strohbehn, 1984, Sminia, Vanderzee, Wondergem, & Haveman, 1994), and no publications have been found that examine short durations of exposure to increased temperatures. In rats, a transient skin and muscle necrosis was observed when the tissues were maintained at 44–45 °C for one hour; while maintaining the spinal cord at a temperature of 42.6 °C for one hour led to no neurological effects, and some ataxia was noted after the spinal cord was held for one hour at 43.0 °C (Franken et al., 1992). The integrated time-temperature of the brain in animals subjected to DTS application does not exceed the time-temperature parameters used in those studies (McLean et al., 2017; Small et al., 2013).

In the current study, when brains were not removed until after carcase processing, a degree of blood vessel engorgement was observed at the proximal tips of the brains. It is unclear whether the blood vessel engorgement is a direct result of energy application alone or hypostatic congestion compounded by post-mortem lividity. Histologically, some areas of lucency were evident immediately below the pia mater, but again it was unclear whether these were processing artefact, associated with the delay between energy application and sample collection, or directly associated with energy application itself.

In the skin pieces, the histological changes observed in the centre of the depilated area are consistent with a second-degree burn as described by Garcia-Espinoza et al. (2017) and it would be expected to heal within 1–3 weeks if the animal had been allowed to recover and return to herd life. Healing could be hastened by using topical burn therapies (Garcia-Espinoza et al., 2017; Liang et al., 2018; Tucker et al., 2014). The temperature profiles at the point of application, however, would suggest that this degree of tissue change would not be present in the conscious animal, as temperatures reached potentially damaging levels late in energy application after it had lapsed into unconsciousness (loss of consciousness occurred between 0 and 5 s after the onset of energy application, based on presence of tonic-clonic response and absence of ocular reflexes), and rapidly fell once energy application ceased. Extrapolation of the temperature declines observed suggests that temperatures would be again below 45 °C within 30–60 s following the end of energy application. During routine processing using DTS, immediately after the waveguide is lifted from the forehead, there is no depilation, and although warmth can be felt when the forehead is touched, it is ‘warm’ not ‘hot’ and the back of the operator’s hand can be laid on the forehead without discomfort. However, after exsanguination, when the body is hoisted onto the processing rail (3–4 min after the completion of energy application), depilation is evident. As these animals were exsanguinated shortly after energy application, we hypothesise that loss of the circulating blood supply to the forehead could have altered the thermal dynamics of the tissue allowing runaway heating to occur during the immediate post-mortem phase. This could also account for the fact that although the forehead hair remains intact when the waveguide is removed after energy application, by the end of the bleed-out phase, depilation can be observed (authors’ observations: data not formally collected).

## 5 Limitations

The study was conducted in a commercial abattoir, producing beef for human consumption. As such, the surgical procedures required to implant fibre optic thermoprobes that would allow direct measurement of temperature changes within the brain or below the skin; or microdialysis probes for measurement of neurotransmitter function, are not feasible: both in terms of animal handling for anaesthesia, and the presence of chemical agent residues in the muscles.

Furthermore, the logistics of processing large species such as cattle results in delays to collection of tissue samples, and this may contribute to the presence of post-mortem change and artefact.

Sample numbers in this study are small, restricted in the case of the temperature measurements by animal ethics approvals. However, the animals processed are representative of the normal operation through the commercial abattoir, and a variety of breeds were included.

## 6 Conclusions

After energy applications of 160 to 200 kJ (18 kW) there were no gross morphological changes to the brain, and limited evidence of changes under on histological examination. Temperatures at the point of application remained close to ambient for the first 8 – 12 s of energy application, then rose to a peak (in all cases less than 60 °C) and began to fall as soon as energy application ceased. Depilation and effects on forehead skin are likely to be exacerbated by post-mortem change. These findings may comply with religious slaughter requirements.

## Funding

This research was funded by Wagstaff Food Services Pty Ltd: Contract number 2022040702.

## Ethical approval

This study was conducted under the authority of the CSIRO ‘Wildlife and Large Animal’ Animal Ethics Committee, reference 2021–05, in accordance with the provisions of the Australian Code for the care and use of animals for scientific purposes (National Health & Medical Research Council, 2013).

## CRediT authorship contribution statement

**Alison Small:** Writing – original draft, Visualization, Validation, Supervision, Project administration, Methodology, Investigation, Funding acquisition, Formal analysis, Data curation, Conceptualization. **Ian Jenson:** Writing – original draft, Visualization, Validation, Formal analysis. **Bruno Fiszon:** Writing – review & editing, Investigation. **Pierre Le Neindre:** Writing – review & editing, Investigation. **Andrew Phillips:** Resources, Project administration, Methodology, Investigation, Conceptualization. **David McLean:** Visualization, Validation, Supervision, Software, Resources, Methodology, Investigation, Data curation, Conceptualization. **Joel McLean:** Software, Investigation, Data curation. **Troy Kalinowski:** Resources, Methodology, Investigation, Data curation. **James Ralph:** Validation, Supervision, Resources, Methodology, Investigation, Funding acquisition, Conceptualization.

## Declaration of competing interest

James Ralph is the commercialiser of the DTS: Diathermic Syncope® technology, funding provider and facilitator of the research. All data collection, data curation, analytical activities and interpretation were performed by the remaining authors, who declare that these activities were conducted in the absence of any commercial or financial relationships that could be construed as a potential conflict of interest.
